# Modeling of nursing care-associated airborne transmission of SARS-CoV-2 in a real-world hospital setting

**DOI:** 10.1007/s11357-021-00512-0

**Published:** 2022-01-05

**Authors:** Attila Nagy, Alpár Horváth, Árpád Farkas, Péter Füri, Tamás Erdélyi, Balázs G. Madas, Aladár Czitrovszky, Béla Merkely, Attila Szabó, Zoltán Ungvári, Veronika Müller

**Affiliations:** 1 Department of Applied and Nonlinear Optics, Wigner Research Centre for Physics, Konkoly-Thege Miklós st. 29-33, Budapest, Hungary; 2 Department of Pulmonology, Semmelweis University, Budapest, Hungary; 3 Environmental Physics Department, Centre for Energy Research, Budapest, Hungary; 4 Envi-Tech Ltd, Budapest, Hungary; 5 Heart and Vascular Centre, Semmelweis University, Budapest, Hungary; 6 1st Department of Pediatrics Semmelweis University, Budapest, Hungary; 7 Clinical Center, Semmelweis University, Budapest, Hungary; 8 Vascular Cognitive Impairment and Neurodegeneration Program, Oklahoma Center for Geroscience and Healthy Brain Aging, Department of Biochemistry & Molecular Biology, University of Oklahoma Health Sciences Center, Oklahoma City, OK 731042 USA; 9 Peggy and Charles Stephenson Cancer Center, Oklahoma City, OK 73104 USA; 10 Department of Health Promotion Sciences, College of Public Health, University of Oklahoma Health Sciences Center, Oklahoma City, OK USA; 11 International Training Program in Geroscience, Doctoral School of Basic and Translational Medicine/Department of Public Health, Semmelweis University, Budapest, Hungary

**Keywords:** Airborne transmission, Aerosol measurement, Lung deposition, Aerosol dispersion, Nursing, Elderly, SARS-CoV-2

## Abstract

Respiratory transmission of SARS-CoV-2 from one older patient to another by airborne mechanisms in hospital and nursing home settings represents an important health challenge during the COVID-19 pandemic. However, the factors that influence the concentration of respiratory droplets and aerosols that potentially contribute to hospital- and nursing care-associated transmission of SARS-CoV-2 are not well understood. To assess the effect of health care professional (HCP) and patient activity on size and concentration of airborne particles, an optical particle counter was placed (for 24 h) in the head position of an empty bed in the hospital room of a patient admitted from the nursing home with confirmed COVID-19. The type and duration of the activity, as well as the number of HCPs providing patient care, were recorded. Concentration changes associated with specific activities were determined, and airway deposition modeling was performed using these data. Thirty-one activities were recorded, and six representative ones were selected for deposition modeling, including patient’s activities (coughing, movements, etc.), diagnostic and therapeutic interventions (e.g., diagnostic tests and drug administration), as well as nursing patient care (e.g., bedding and hygiene). The increase in particle concentration of all sizes was sensitive to the type of activity. Increases in supermicron particle concentration were associated with the number of HCPs (*r* = 0.66; *p* < 0.05) and the duration of activity (*r* = 0.82; *p* < 0.05), while submicron particles increased with all activities, mainly during the daytime. Based on simulations, the number of particles deposited in unit time was the highest in the acinar region, while deposition density rate (number/cm^2^/min) was the highest in the upper airways. In conclusion, even short periods of HCP-patient interaction and minimal patient activity in a hospital room or nursing home bedroom may significantly increase the concentration of submicron particles mainly depositing in the acinar regions, while mainly nursing activities increase the concentration of supermicron particles depositing in larger airways of the adjacent bed patient. Our data emphasize the need for effective interventions to limit hospital- and nursing care-associated transmission of SARS-CoV-2 and other respiratory pathogens (including viral pathogens, such as rhinoviruses, respiratory syncytial virus, influenza virus, parainfluenza virus and adenoviruses, and bacterial and fungal pathogens).

## Introduction

Coronavirus disease 2019 (COVID-19) caused by infection with the novel coronavirus SARS-CoV-2 (severe acute respiratory syndrome—coronavirus 2) resulted in a deadly pandemic [1]. COVID-19 has caused more than 5.3 million deaths worldwide to date [2]. Importantly, SARS-CoV-2 infection causes worse outcomes (e.g., increased risk of hospitalization, intensive care unit admission, and invasive mechanical ventilation) and results in a significantly higher mortality rate in older adults [1]. Most of COVID-19 deaths in the USA have been among adults 65 years and older [1, 3].

The principal mode of infection is through exposure to respiratory fluids carrying infectious SARS-CoV-2 virus particles [4]. There is strong evidence that transmission of SARS-CoV-2 may occur through close contact with an infected person through airborne particles (including both respiratory droplets and aerosols) [4, 5]. The view is emerging that infected people, by breathing, talking, sneezing, and coughing as well as by medical procedures, release airborne particles of different sizes consisting of respiratory fluids carrying an infectious dose of viable SARS-CoV-2 [4, 5]. While the larger droplets settle out of the air rapidly, the smallest droplets and fine aerosol particles remain suspended in the air for an extended period of time [4, 5]. SARS-CoV-2 is transmitted by inhalation of air carrying airborne particles of different sizes that contain infectious virus [4, 5]; by deposition of these virus-loaded particles onto exposed mucous membranes (e.g., in the nose, mouth, and eye) and by touching mucous membranes with the hands soiled by exhaled respiratory fluids (including exhaled virus-carrying particles settled on inanimate surfaces) [4, 5]. Current research projects focus on several aspects of these processes, from understanding the physics of the formation and deposition of airborne particles and to identifying factors that impact their size and concentrations. To date, multiple physiological and environmental factors have been identified, which likely affect the respiratory transmission of SARS-CoV-2. Among them, crowding and sharing common facilities as well as indoor settings with inadequate ventilation or air handling are known to modulate the concentration of airborne particles and are thus risk factors for transmission of SARS-CoV-2 [6]. Other critical factors that determine the risk of respiratory transmission of SARS-CoV-2 include the distance from the source, increased exhalation by the infected person (e.g., during physical exertion and singing), air flow physics, and length of exposure [4]. The dynamic processes influencing the movement and size of the aforementioned airborne particles are of special importance. Observing and monitoring the presence and transport of airborne particles is particularly important in closed spaces, such as hospital rooms.

Older adults living or staying in congregate settings, such as nursing home bedrooms and hospital rooms, are at an especially high risk of respiratory transmission of SARS-CoV-2 by airborne mechanisms. These are typically enclosed spaces without effective removal of airborne particles where patients stay for an extended period of time. Moreover, older nursing home residents are frequently frail with a dysregulated immune function (immunosenescence [6–8]) that contributes to their increased susceptibility to infection [1, 9–11]. As a result, the COVID-19 pandemic has devastated nursing homes in the USA [12–15] as well as the European Union [16, 17]. Although only around 0.4 to 0.7% of the total population of the USA and the European Union live in nursing and residential care facilities, long-term-care facility deaths have accounted for ~ 25 to 74% of the documented deaths due to COVID-19 in many regions on both contients [14, 18, 19]. In the USA, over 8% of people who live in long-term-care facilities, which include nursing homes, assisted living and other long-term care facilities, have died of COVID-19 (for nursing homes alone, the figure is close to 10%) [20]. Before COVID-19, nursing home associated respiratory infections already represented an important health challenge [21–25].

Healthcare professionals (HCPs) are essential workers defined as persons serving in health care settings who are directly or indirectly exposed to patients with COVID-19 and potentially to infectious materials [26–28]. There is strong evidence that HCPs could play a significant and multifaceted role in pathogen transmission [29, 30].

The present study was designed to determine how the daily activities of HCPs impact the concentration and size distribution of airborne particles in a hospital room housing an elderly patient with COVID-19. To achieve that goal, an optical particle counter was placed (for 24 h) in the head position of an empty bed in the hospital room of a confirmed COVID-19 patient admitted from the nursing home. The type and duration of the activity, as well as the number of HCPs providing patient care, were recorded. Concentration changes associated with specific activities were determined. The measured data were also used to quantify the associated risks by applying a lung deposition model of the inhaled particles for individuals needing full medical/nursing support. HCP activities associated with the highest risk of possible airborne particle mediated infection were identified and characterized.

## Methods

**Fig. 1:**
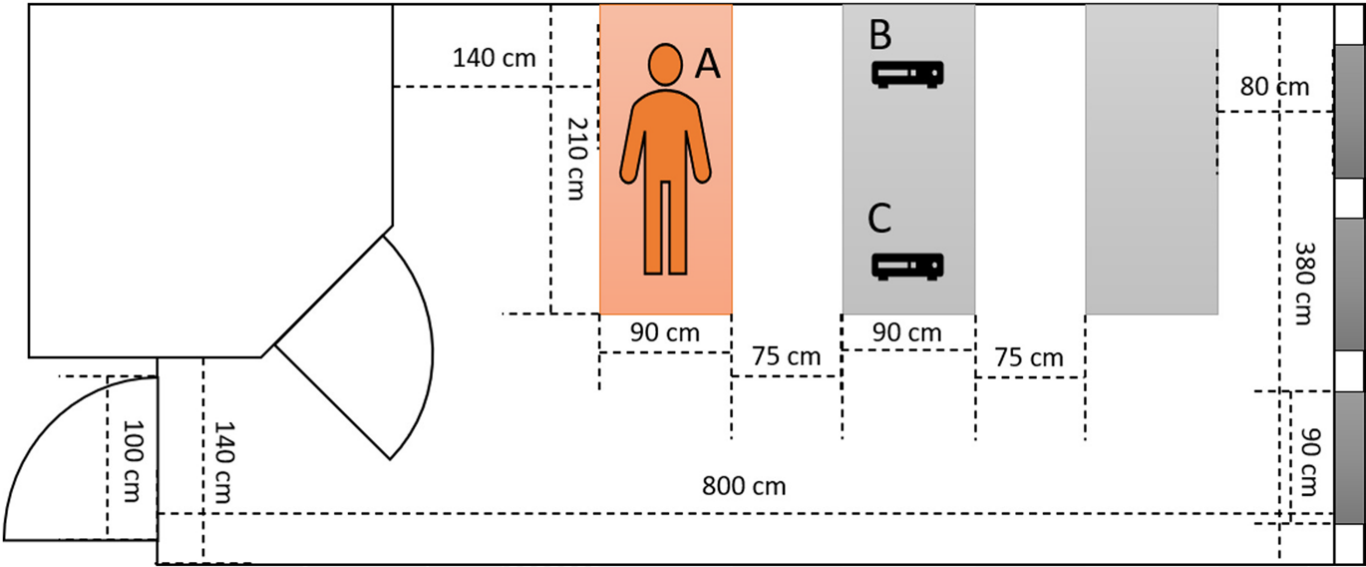
The layout of the three-bed patient room. A Patient with COVID-19 confirmed with PCR test. B Optical particle counter located at the head position of an imagined patient. C Optical particle counter at the foot position of an imagined patient

Measurements were performed over a 24-h period in a closed patient room (Department of Pulmonology, Semmelweis University, Budapest, Hungary) with one PCR positive COVID-19 patient admitted from a nursing home during the first wave of the pandemic (March–June 2020). This three-bed patient room was selected as the patient was unable to leave the bed needing full medical and nursing support. The layout of the patient room is depicted in Fig. 1. There was no heating, ventilation, and air conditioning in operation due to local public health regulations. The patient room was thoroughly cleaned to prevent resuspension of previously deposited airborne particles. The patient could not tolerate wearing a mask. HCPs wore appropriate personal protective equipment (PPE) all the time, including single-use plastic-based protective clothing, face shields, and N95 respirators, which prevented both HCP exposure to respiratory droplets and airborne particles and release of respiratory droplets and airborne particles by the HCPs.

All HCP activities in the room were recorded as follows: activities and number of personnel as well as time spent in the room. Measuring devices were placed on the empty middle bed in the head and feet positions. The measurements were repeated at a later time point in the same room when no patient was present for 24 h to assess background particle concentrations, confirming stable values during the day without human activity.

The events corresponding to the activities were classified into three categories: A) physician and/or nurse visit for medical diagnostic examinations (e.g., electrocardiogram and ultrasound) and/or therapy administration (e.g., infusion and medication); B) nursing care (e.g., feeding, personal hygiene, bedding, and full patient care); C) patient’s activity. While A and B were logged by the medical staff, C was added by post-processing of the data. Aerosol particle concentrations (number/liter) and size distributions were measured by an optical particle counter (OPC—Grimm Aerosoltechnik, Portable Aerosol Spectrometer, model 1.109) for 24 h with 1-min time resolution. The size distributions were recorded in 31 size bins between 0.25 and 32 µm. The upper concentration limit (< 5% coincidence error) of the instrument is 2 million particles/l. For the measurements, we used the radial symmetric sampling head provided by the manufacturer of the OPC. The device can be operated autonomously. It is small (24 × 13 × 7 cm^3^) and quiet, making it suitable for the measurements in a hospital room for a longer time without disturbing the patient and the medical personnel. Concentrations corresponding to the events were calculated by averaging the measured numbers for the events’ duration. The baseline concentration was determined by averaging the data from the preceding 5 min of each activity.

The airway deposition of the particles with the measured size distributions corresponding to the selected activities was simulated by the stochastic lung deposition model [31–33]. The computations refer to particle inhalation without a mask. Nose breathing was assumed with 500 ml tidal volume, 2100 ml functional residual capacity, 1.9 s inhalation, 0.1 s breath-hold between inhalation and exhalation, 2.0 s exhalation, and a 1.0 s breath-hold after the exhalation, representing quiet breathing at rest (12 breaths/min). The deposition model simulates the airways’ asymmetric branching system and provides the fraction and density of particles deposited in different anatomical regions. In order to calculate the density values, the surface of different regions is needed. The extrathoracic airways representing the nasopharyngeal cavity have a relatively small surface (set at 0.047 m^2^). The area of conductive airways from the trachea till respiratory bronchioles, including terminal bronchiole, is smaller (set at 0.47 m^2^) compared to the acinar surface (set at 147 m^2^) [34]. The model has been validated against experimental data as published previously [35, 36].

## Results

Concentration values measured by the two OPC devices revealed that there was no significant difference at the two sampling locations. Separate day background measurements in the same unoccupied room showed that the aerosol particle concentration and size distribution did not change in the absence of human activity.

**Fig. 2:**
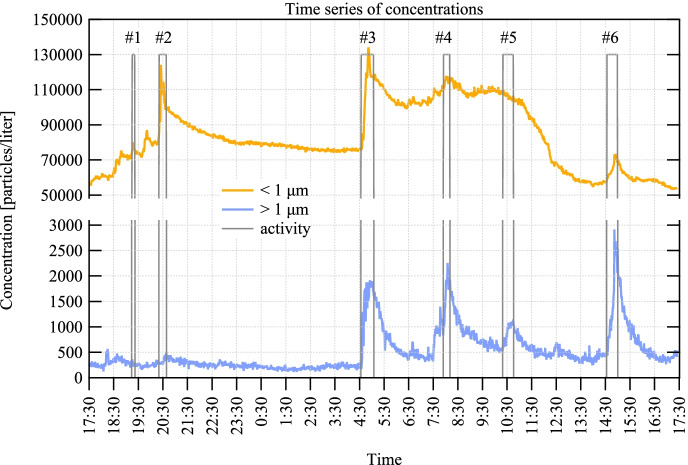
Time series of the measured number concentrations in two size bins, below 1 µm (orange curve) and above 1 µm (blue curve) with 1-min time resolution. The grey rectangles show the six selected events indicating the duration of the activities by their width

Figure 2 shows the time dependence of the concentrations in the sub- and supermicron size ranges in the real-world setting representing different activities corresponding to HCP and patient-generated particle peaks. It can be seen that the total aerosol particle concentration in the hospital room varied between 55,000 and 135,000 particles/l in one day.

Altogether, thirty-one events were logged during the 24 h’ period, from which six representative ones were selected in the current study. These six events represent all categories (A, B, and C) and correspond to well-defined peaks on the concentration trends graph (Fig. 2 and Table 1).

**Table 1:** Description of the selected events with measured baseline concentrations and the events’ contributions to the baseline (average number of aerosol particles/l)

Event No	Category	No. of personnel	Duration [min.]	Baseline < 1 µm	Contribution < 1 µm	Baseline > 1 µm	Contributio > 1 µm
1	C	0	6	71,457	5843	274	0
2	A	1	16	79,665	26,136	281	55
3	B	2	30	76,204	33,142	213	1229
4	A	2	15	110,835	3682	1018	658
5	A	3	28	108,454	0	552	346
6	A and B	3	25	57,295	7997	444	1019

The measured size distributions can be modeled by a double peak log-normal distribution. Based on the size bins’ contributions to the two modes of the distribution, the instrument’s size range can be split into two parts at 1 µm diameter, where the particles’ properties and their generation mechanisms are significantly different. The majority of the activities in a hospital room contribute to both modes (size ranges) to different extents. Cleaning, bedding, or other significant movements by HCPs contribute more to the increases in supermicron sized particles reaching the measurement device.

It can be observed in Fig. 2 that the maxima in the sub-micron and supermicron size ranges do not always overlap. For events #2, #3, and #4, the sub-micron peak occurs in the first or middle part of the event duration, while in the range above one micron, the maximum (where present) is reached by the end of the event period, which we attribute to air flow dynamics. As soon as the activity ceases, the concentration decreases in the supermicron size range. In event #6, both maxima occur towards the end of the period; however, there is also a 3–4-min-long difference between them. There was no increase in the concentration above one micron for event #1 and below one micron for event #5 associated with the given activities, which might represent differences in particle distribution and disturbance related to specific activities.

In Fig. 2, activities #3 and #4 seem to lead to a much slower particle concentration decay than the others. This is explained by the fact that there were other activities shortly after activities #3 and #4, which are not shown in the figure, but also affected the particle count.

In general, the larger the number of sampled particles during a given activity, the longer the time of particle concentration decrease to the pre-activity level measured at the position of a neighbor bed patient’s head. Table 1 shows the activities’ contributions to the baseline concentrations during the six selected events, together with their category, duration, and the number of HCPs in the room.

**Fig. 3:**
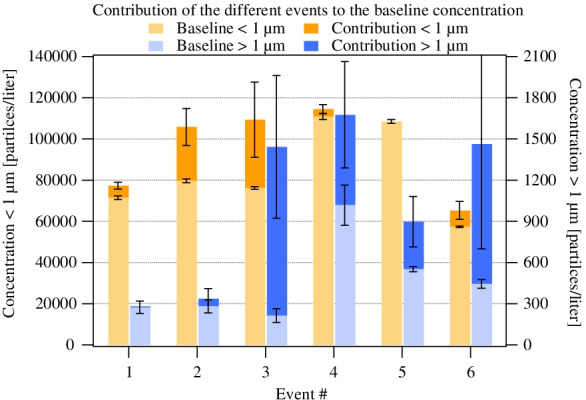
Contributions on top of the baselines in the two size bins. The baselines are the concentrations preceding the events. The contributions were calculated by subtracting the baseline concentrations from the measured concentrations during the events

Figure 3 shows the events’ contributions in the two size ranges on top of the corresponding baseline concentrations. It can be seen that the different events result in different patterns of the contribution added to the baseline value in the two selected size ranges.

For events #1 and #2, the supermicron contribution is negligible, while it is significant for events #3, #4, #5, and #6. For events #4, #5, and #6, the supermicron contributions are comparable or larger than the baseline, while the sub-micron contributions are relatively small. For event #3, there are significant increments in the concentrations in both size ranges. As our results demonstrate, there is a significant dependence of the particle number on the type of activity. HCP activities related to physician and/or nurse visits for medical diagnostic examinations and nursing care are among the most important events causing the increase in the number of submicron particles. By the same token, activities related to full patient care, but especially bedding, have contributed significantly to the rise of larger particles’ concentration in the air.

Besides the type of activity, the concentration of particles may depend on the number of personnel carrying out the given activity and its duration. Our analysis has shown that the increase of submicron particles as a result of different patient and HCP activities (all the monitored activities, not only those selected and marked in Fig. 2) did not correlate with the number of personnel inside the hospital room and correlated only weakly and insignificantly (*p* > 0.05) with the duration of the activity. In contrast, the increase of the number of larger particles (> 1 µm) fairly correlated with the number of HCPs in the room (*r* = 0.66, *p* < 0.05), and a significant correlation was found with the duration of the activity (*r* = 0.82, *p* < 0.05).

**Fig. 4:**
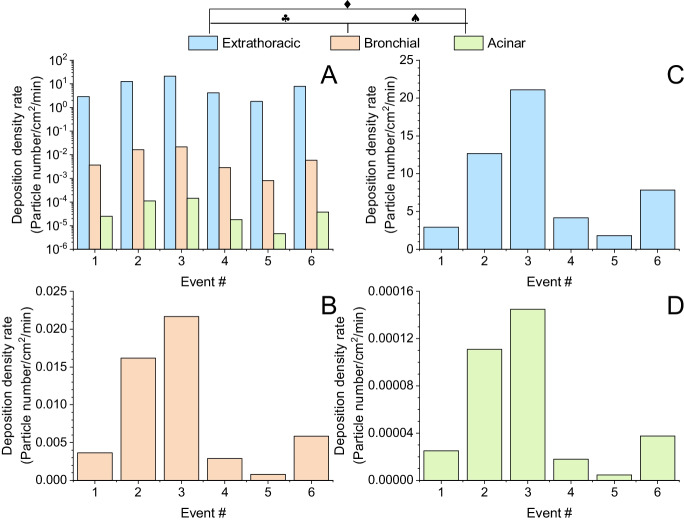
Extrathoracic, bronchial, and acinar deposition density rates of the number of inhaled aerosol particles in the studied patient room. **A** All anatomical regions. **B** Extrathoracic airways. **C** Bronchial region. **D** Acinar region. Note the logarithmic vertical scale of the top-left summary plot. Calculated Pearson correlation coefficients with two-tailed test of significance: ♦*r = *0.972,** p < **0.05; ♣*r = *0.977,** p < **0.05; ♠*r = *0.999,** p < **0.05

The results of the numerical simulations of the deposition density rates (particle number/cm^2^/min) of the inhaled particles within different anatomical regions of the airways are summarized in Fig. 4. The majority of the measured particles were in the range of a few hundred-nanometer diameters. As a result, the number of particles deposited in unit time was the highest in the acinar region. Number deposition density rates were on average 1.5 × 10^5^ times higher in the extrathoracic airways than in the lungs (bronchial + acinar).

## Discussion

The main finding of the study is that HCP activity exerts significant effects on hospital patient room aerosol particle size and concentration distribution.

Nursing home patients are at the highest risk of SARS-CoV-2 infection, especially when sharing rooms with roommates of undiagnosed infection [14]. Individual patient rooms could lower the risk of infection and early screening plays a significant role in controlling disease spread. The present setup was designed for nursing care or critically ill patients needing full medical and nursing care. We performed aerosol change measurements in a real-world hospital setting. Our measurements were performed on the adjacent bed, where a potential roommate could inhale from the room air particles released by the other patient and disturbed by different HCP activities [27]. It is important to emphasize that activities requiring at least 2 HCPs (eg., caring for obese patients) were associated with higher particle concentration.

Once airborne particles carrying the SARS-CoV-2 virus are exhaled, the risk for infection of a roommate is influenced by several factors, among which the concentration of airborne particles carrying the virus and their deposition in the respiratory system are of special importance. We found that daytime activities of HCP and patients are resulting in a constant increase in the number of submicron particles, whereas supermicron particle number changes represent more distinct peaks followed by a rapid decay. Nighttime without significant activity was associated with a low-level concentration in sub- and supermicron aerosol particles.

There are many known medical interventions (e.g., tracheal intubation, tracheotomy, cardiopulmonary resuscitation, bronchoscopy, and sputum induction) that result in significant increases in the generation and release of airborne particles [37, 38]. Recent studies show that among the aerosol-generating procedures, high-flow oxygen therapy and non-invasive ventilation are also associated with a significant increase in the release of airborne particles and consequential viral spread around patients in need of these therapeutic interventions [39].

While the focus of our study was the characterization of particle generation and deposition associated with the activities of HCP, we also identified particle emissions that were attributed to the patient’s activity. It is now widely recognized that individuals emit particles during coughing, sneezing, loud speaking, singing, or normal breathing [40]. While the amount of exhaled particles varies several orders of magnitudes between human subjects, it has been shown in a recent study [41] that individuals can be categorized into two distinct groups by means of aerosol particle emission rate. The high-producers (18% of the examined group) were responsible for 80% of the exhaled particles, while the low-producers emitted a significantly lower amount. The inhalation of isotonic saline can significantly reduce the number of exhaled particles among the high-producer subjects for up to 6 h after inhalation [42]. These results suggest that the particle emission of C category events (patient’s activity, coughing, sneezing, etc.) might be reduced by inhalation of isotonic saline, reducing the associated risks of airborne infections of roommates. Air movements caused by the opening of doors and windows might also influence the concentration and deposition of airborne particles in hospital and nursing home settings.

Particles inhaled from the room air deposit in different parts of airways and lungs, and if carrying the virus, this contributes to the spread of COVID-19 among hospitalized or nursing home residents [43]. How particles deposit is determined by the size, composition, concentration, and coagulation of the particles as well as the influence of external factors (e.g., physical forces, such as air movement). In line with previous results [44], larger particles deposit in the upper airways, while small particles enter acinar regions of the lungs. Deposition density was higher for the acinar region during patient activities (might include cough, position change in the bed, etc.).

Airway deposition simulations demonstrated that the number of particles depositing in the airways and the probability of infection depends on the type of activity. In addition, the distribution of particles among different anatomical regions is also a function of the activity type. We found that HCP activities are predominantly associated with changes in supermicron airborne particles, which deposit mainly in the upper airways. Similarly, higher physical activity in classrooms or offices also affects larger airborne particles. Activities generating a higher number of small particles lead to higher lung acinar deposition.

Generally, the higher the concentration of the particles associated with a certain activity, the higher the probability of infection. Simulations show that for one cm^2^ of airway epithelium, more particles are deposited in the extrathoracic airways. This can explain why frequent early symptoms of the SARS-CoV-2 are sore throat and loss of taste and smell [45]. These results also suggest the opportunity for oral administration of aerosolized disinfectants, antiviral treatment, or even corticosteroids with smaller particle sizes (ciclesonide and beclomethasone) to avoid severe disease [46]. The data suggest that a bedridden roommate’s activities increase airborne particles in the sub-micron range, which likely promote the acinar entry of possible infective agents. This might partially explain the rapid spread of COVID-19 pneumonia in nursing homes and hospital settings which still represents a high risk for unvaccinated individuals [19].

The actual amount of virus to which a roommate is exposed is also influenced by the virus content of the individual particles, the decreases in the concentration of virus-carrying airborne particles (e.g., due to falling of larger respiratory droplets to the ground) and loss of viability and infectiousness of the virus carried by the airborne particles due to high temperature, and ultraviolet radiation (e.g., sunlight).

### Limitations of the study

Airborne particle is a general term for all the measured sub-visible matter suspended in the air. Indoor air contains a variety of different airborne particles, which cover a spectrum of different sizes. In addition to respiratory droplets and particles emitted from the patient, these also include particles of dust and dirt as well as other microorganisms. Studies conducted in cleanrooms demonstrated that personnel are the main contributors to particles. It is a major limitation of our study that we did not have the opportunity to assess the physical properties or the virus content of the airborne particles. Furthermore, the optical particle counter we used in this study is size-calibrated with PSL particles, which is a widely accepted calibration method in case of general airborne particles. Although the optical properties of the exhaled particles generated in the lungs differ from the optical properties of PSL spheres, they may stick to other airborne particles producing a mixture with barely predictable optical properties, which may affect the cut-off diameters of the instrument, resulting in slightly distorted size distributions. However, this limitation does not affect our conclusions. Thus, further studies are warranted to assess the effects of HCP activity on actual viral transmission by very small fine respiratory droplets and aerosol particles that contain an infectious virus.

We also did not have the opportunity to record voice and images, with which the description of the patient’s activities could have been refined. It is important to note that in the case of patients needing oxygen therapy, the constant flow of the therapeutic gas might significantly influence particle distribution. Additionally, changes in the distance between roommates can also influence aerosol particle traffic and deposition but were not measured as a variable. Additional measurements are also needed to evaluate external influences in nursing home or hospital room settings.

#### Conclusions

Our experimental setup made it possible to closely monitor the interaction between HCPs and a COVID-19 patient in a hospital ward and its effect on environmental airborne particle changes. Our data confirm the need for more data describing these changes, especially as they may be crucial for a better understanding of nursing home or hospital-acquired airborne transmission of SARS-CoV-2, a well-known but not fully understood safety issue in the care of the elderly. A better understanding of the generation, release, and movement of airborne particles in a closed room leads to a better understanding of how potentially virus-carrying particles contribute to airborne transmission of SARS-CoV-2 in hospitals and long-term care facilities. Gaining this knowledge also provides the potential to develop new methods to prevent the release and/or effectively remove these airborne particles and establish a healthier air environment.

Personal protective equipment and strict protocols aim to minimize the chance of infection of HCP and other individuals [47, 48]. Social distancing could contribute to lowering the spread of airborne transmitted diseases, including COVID-19 [49], but in nursing homes and other long term care facilities and hospitals, its implementation proved to be challenging. Proper air filtration and adequate control of air movement are also essential to prevent airborne transmission of SARS-CoV-2 in hospital rooms and long term care facilities housing older individuals at risk of COVID-19 infection. In the early phase of the pandemic, negative pressure patient rooms were optimal to protect HCPs from infection [50]. In contrast, positive pressure rooms are used for severely immunocompromised patients to protect them from infections. In this case, the positive pressure might press the particles down close to the floor and protect the individual from inhaling the invisible particle disturbance caused by the treating medical staff [51]. Measures, including the optimal positioning of patient beds, proper ventilation of hospital rooms, and minimizing of the activities that significantly increase the risk of infected particle inhalation, need to be investigated. Air exchange rates should also be monitored and controlled. Adequate air changes dilute potential virus-carrying airborne particles to an acceptable concentration. It is also very informative to determine the time needed for a patient room to return to the static condition with acceptable airborne particle concentration following a high respiratory particle generating event.
